# Calcific bursitis of the Gruberi bursa: a case report

**DOI:** 10.1186/s13256-024-04377-7

**Published:** 2024-02-17

**Authors:** Nikhil N. Patel, Jean Jose, Cristina Pravia

**Affiliations:** University of Miami Miller School of Medicine, Miami, FL USA

**Keywords:** Calcific bursitis, Gruberi bursa, Ankle swelling, Non-operative, Case report

## Abstract

**Background:**

Bursitis is the inflammation of a synovial bursa, a small synovial fluid-filled sac that acts as a cushion between muscles, tendons, and bones. Further, calcific bursitis results from calcium deposits on the synovial joint that exacerbates pain and swelling. The Gruberi bursa is located dorsolaterally in the ankle, between the extensor digitorium longus and the talus. Despite limited literature on its pathophysiology, the aim of this case is to discuss the bursa’s association with calcific bursitis and its management via a case presented to our clinic.

**Case presentation:**

A 47-year-old Caucasian female with no past medical or family history presents with acute right ankle pain following a minor injury 3 months prior with no improvement on analgesic or steroid therapy. Imaging demonstrated incidental calcium deposits. The day prior to presentation, the patient stated she used 1-pound ankle weights that resulted in mild swelling and gradual pain to the right dorsoanterior ankle. Physical exam findings displayed a significant reduction in the range of motion limited by pain. Imaging confirmed calcification within the capsule of the talonavicular joint, consistent with Gruberi bursitis. Initial management with prednisone yielded minimal improvement, requiring an interventional approach with ultrasound-guided barbotage that elicited immediate improvement.

**Conclusion:**

The presented case report highlights a rare and unique instance of acute ankle pain and swelling caused by calcific Gruberi bursitis in a young female. Although the Gruberi bursa is a relatively new discovery, it contains inflammatory components that may predispose it to calcification and should be considered in the differential of ankle swelling. Therefore, utilizing a systematic approach to a clinical presentation and considering all differential diagnoses is essential.

## Background

Ankle pain and swelling is a common clinical presentation resulting from various conditions, including sprains, gout, and calcific periarthritis. A synovial bursa is a small sac filled with synovial fluid that acts as a cushion between muscles, tendons, and bones [1]. Bursitis is a common musculoskeletal presentation of an irritated, swollen, or infected bursa capsule that elicits pain and reduces range of motion [2, 3]. The four most common locations for bursitis are prepatellar, olecranon, trochanteric, and retrocalcaneal; typically, these respond to non-surgical management [4]. Calcific bursitis is the build-up of calcium deposits in soft tissue, which can result in painful swelling of the bursa in synovial joints such as the shoulder, elbow, fingers, wrist, hip, knee, and, less commonly, the ankle [5]. In this report, we present a case of acute onset ankle pain and swelling secondary to calcific Gruberi bursitis requiring interventional non-operative management.

First described by Alexander Monro (1825), the Gruberi bursa is located between the extensor digitorum longus (EDL) tendon and talus [6]. The Gruberi bursa is found on the dorsolateral ankle. There is limited literature describing its pathophysiology and its relationship with calcific bursitis.

## Case presentation

**Fig. 1.**
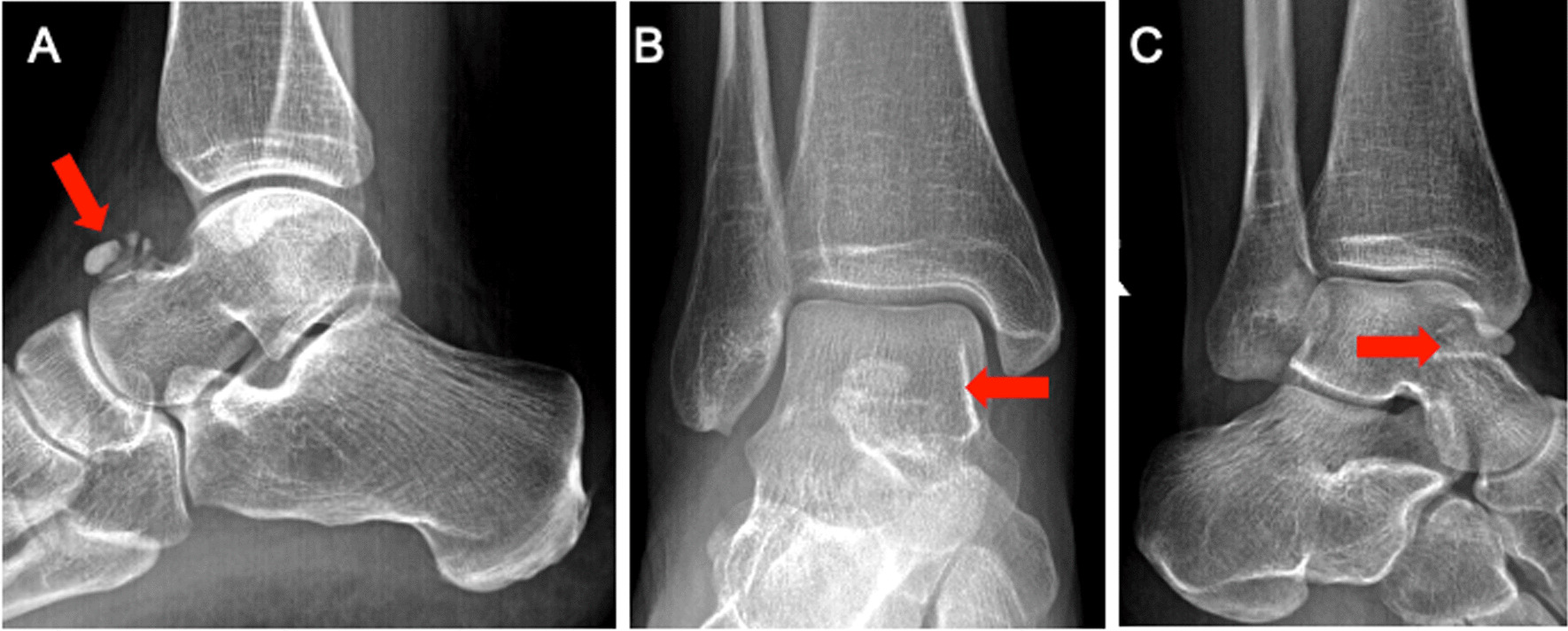
A 1.4 × 0.9 × 1.1 cm focus of soft tissue calcification, with juxta-articular deposits of calcium hydroxyapatite along the dorsal aspect of the talus (arrows)

A 47-year-old Caucasian female with no past medical or family history presented to the clinic for evaluation and treatment of 1-day acute right ankle pain. The patient stated that she sustained a minor injury 3 months prior when she tripped and fell, hyper-plantarflexing the right ankle; she endorsed pain toward the base of her toes rather than her ankle. A plain X-ray of the right ankle in anteroposterior (AP) and lateral noted “incidental calcium deposits over the talonavicular region” (Fig. 1).

Subsequently, she went to a pilates class and used 1-pound ankle weights for the first time. The next day, she woke up with mild swelling and gradual, progressive pain to the right dorsoanterior ankle. She was unable to ambulate or bear weight on that foot within 24 hours of the pilates session. The patient denied fevers, chills, rashes, or previous history of rheumatological disorders. Upon physical exam, the right ankle showed noticeable swelling to the anterior aspect without erythema or warmth. Dorsalis pedis was palpable, and strength was 4/5 in all muscle groups of the right leg. The pain was localized predominantly to the anterior aspect of the ankle centrally and over the talonavicular region and tender at the extensor digitorum commonness. The Achilles, posterior ankle, tibialis anterior, and extensor hallux longus tendons were non-tender. Sensation was intact to light touch. Range of motion was severely limited by pain.

**Fig. 2:**
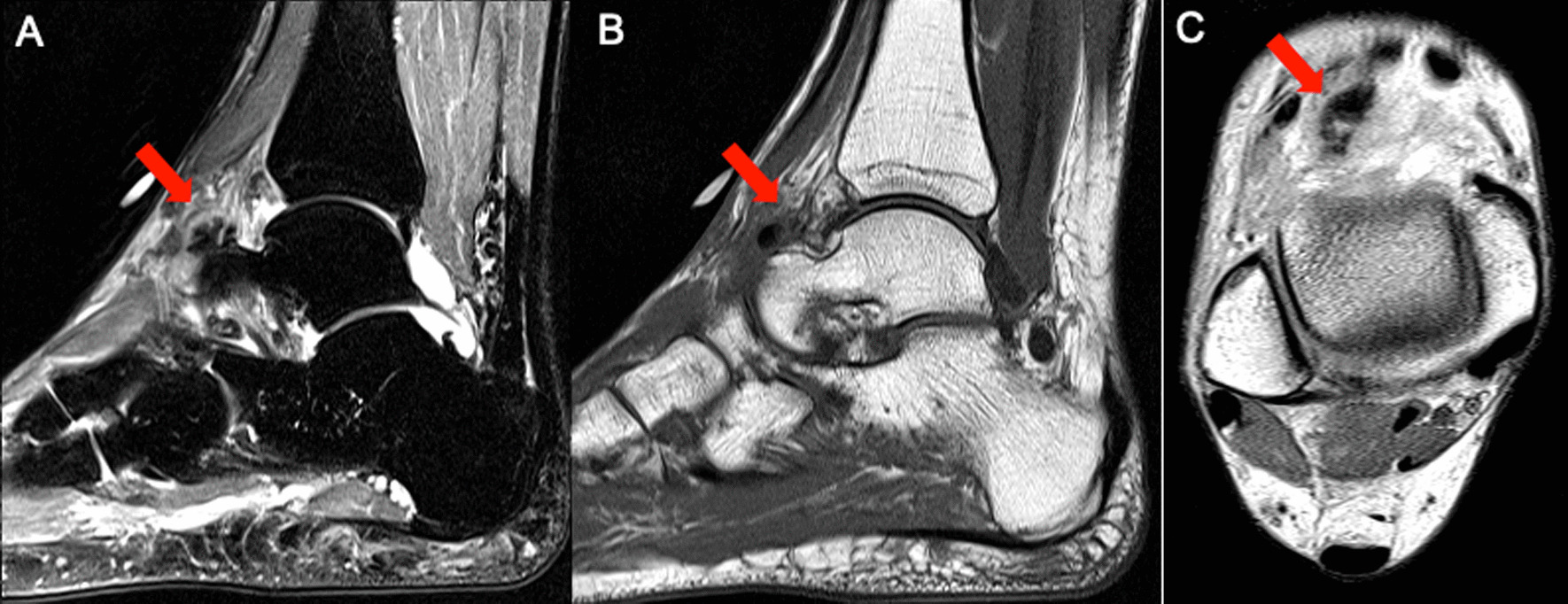
(**A**) Sagittal Short Tau Inversion Recovery, (**B**) Sagittal T1, and (**C**) Axial Proton Density Magnetic Resonance Images demonstrate a calcified soft tissue mass with surrounding inflammatory changes along the dorsal aspect of the talus, involving the inferior extensor retinaculum (frondiform ligament) as it passes along the extensor digitorum tendons, reflecting calcific Gruberi bursitis (arrows)

Repeat X-ray at this acute presentation demonstrated no evidence of fracture or osteochondral pathology but again showed a calcification dorsal to the talonavicular region. This appeared different than an ossicle or osteophyte, providing concern for acute calcific periarthritis. A magnetic resonance imaging (MRI) of her ankle did not show any soft tissue masses but confirmed calcification within the capsule of the talonavicular joint without involvement of the extensor tendons. Using the MRI findings, the precise anatomical location was determined to be consistent with Gruberi bursitis (Fig. 2). She was prescribed prednisone 40 mg daily for 4 days without significant improvement.

**Fig. 3:**
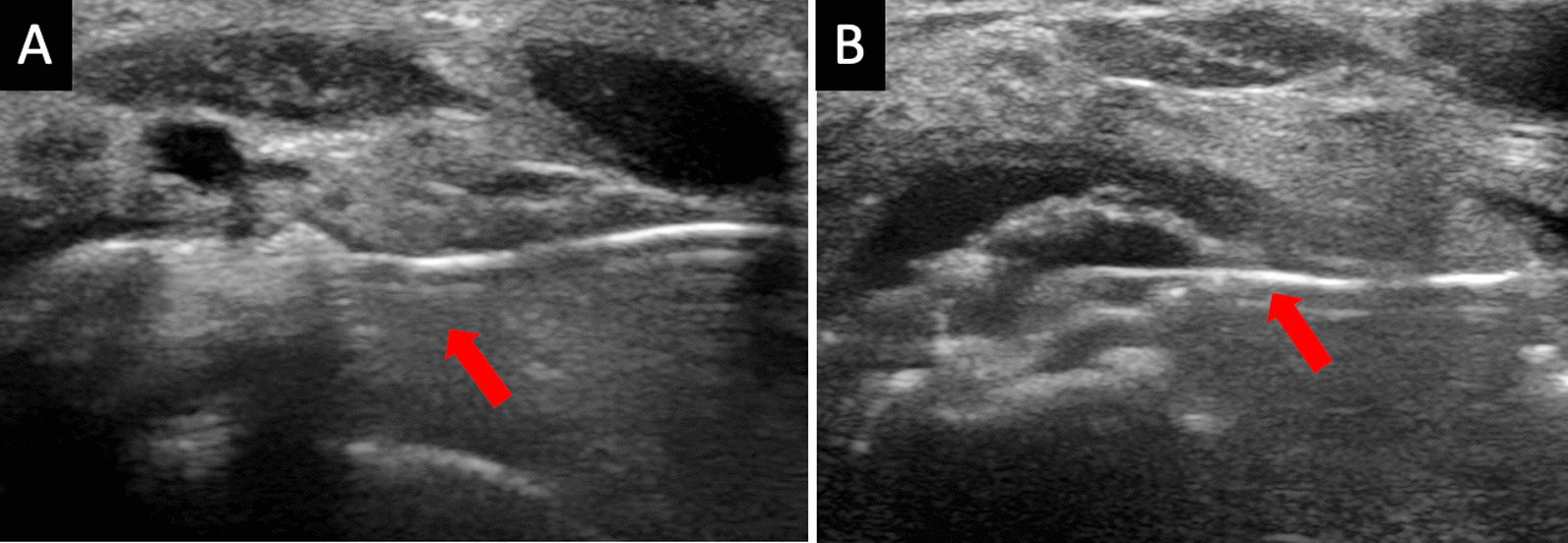
(**A**), (**B**) Longitudinal grayscale ultrasound images of the ankle demonstrate ultrasound-guided calcific bursitis lavage using a 20-gauge spinal needle (arrows)

**Fig. 4:**
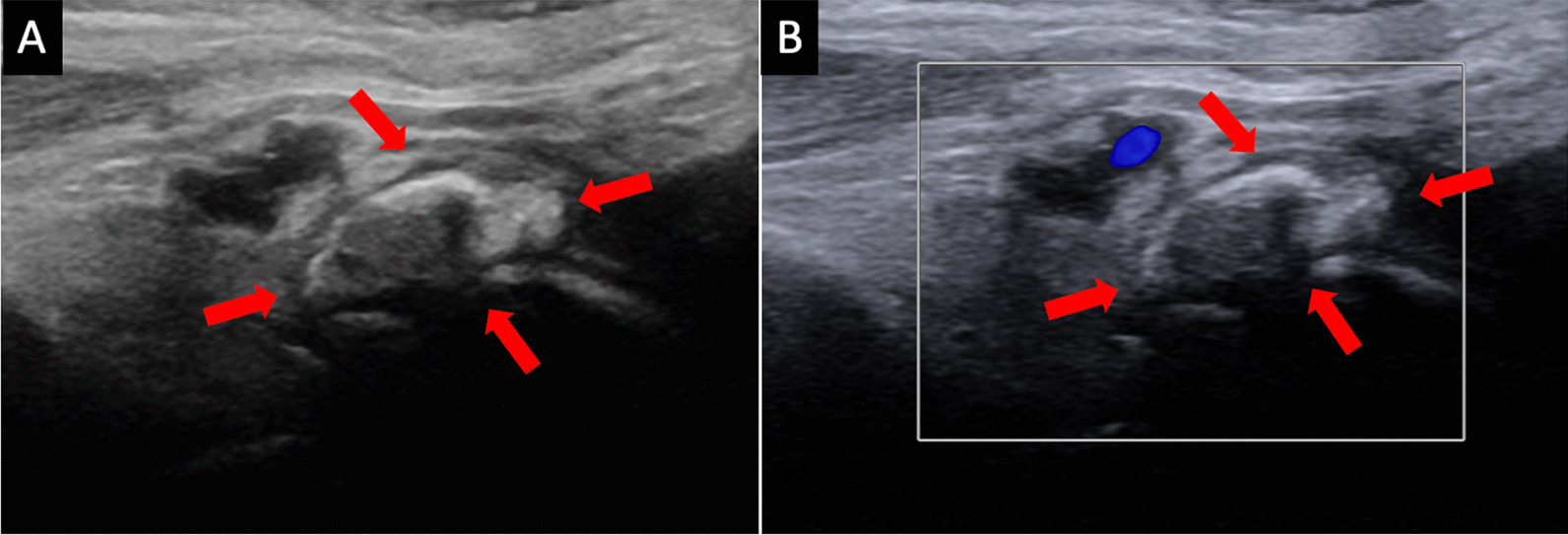
(**A**) Longitudinal grayscale and (**B**) color Doppler ultrasound images of the ankle demonstrate hyperechoic foci with acoustic posterior shadowing, internal calcific content, and no appreciable internal vascularity (arrows)

Upon consultation with interventional radiology, an ultrasound (US)-guided barbotage was performed (Fig. 3). The inflamed right ankle Gruberi bursa was identified, and amorphous calcifications were noted dorsally to the talus, measuring 1.4 × 0.6 × 1.8 cm, consistent with calcific bursitis (Fig. 4). A 20-gauge needle was advanced into the right foot, and a mixture of lidocaine and saline was injected with multiple passes through the amorphous calcifications, breaking them down enough to be lavaged out. Subsequently, a mixture containing 1 cc (40 mg) of Kenalog and lidocaine was injected into the area of calcific bursitis. Within minutes, the ankle pain significantly improved [Numeric Pain Rating Scale (NRS) pain pre-procedure 10/10 and post-procedure 2/10], and the patient immediately returned to baseline ambulation. Rest, ice, and anti-inflammatories were recommended on discharge. Upon 6-month follow-up, this patient is endorsing no pain in her ambulation nor at rest.

## Discussion

Despite mentions of this anatomical bursa in early twentieth-century anatomy textbooks and multiple reports using MRI or US, extensive debate exists on whether the Gruberi bursa communicates with the talonavicular joint, the tibiotalar joint, and the EDL tendon [1, 7–10].

In this report, we present a unique case of Gruberi bursitis as a rare cause of acute dorsolateral ankle pain and swelling in a young female. While calcific bursitis can affect various bursae in the body, no such literature exists, with only Ragab *et al.* discussing MRI findings of Gruberi bursitis [1].

Calcific bursitis presents with localized pain, swelling, tenderness, and reduced range of motion that can worsen with activity, repetitive trauma, or chronic irritation and deposit formation [11, 12]. Common management includes rest, immobilization, nonsteroidal anti-inflammatory drugs (NSAIDs), and physical therapy to reduce pain and inflammation [13]. Ice and compression can further alleviate associated symptoms. In consultation with orthopedic surgery, operative management, including arthroscopic removal of the calcific deposit, excision of the bursa, or performing a bursectomy, can be considered if conservative treatment fails to improve the bursitis. Interventional radiology measures, as in our case, can also be performed to aspirate the calcification and inject corticosteroids for pain and inflammation management. Barbotage is a low-risk procedure requiring local anesthesia and relatively quick recovery. The improvement seen in this patient thus supports the belief that inflammatory bursitis to the Gruberi bursa due to calcific involvement exists.

A common differential diagnosis for calcific bursitis is acute calcific periarthritis [14–16]. Although periarthritis is also caused by calcium deposits within soft tissue, resulting in rapid onset monoarticular pain, swelling, erythema, or fever, the cardinal difference is the involvement of tendons [14, 17–19]. Similarly, calcific periarthritis is found in big joints such as the shoulder and is self-resolving or requires conservative treatment [15]. Our case represents a unique presentation in the ankle that required alternative therapy. Despite high doses of oral steroids, the patient was non-ambulatory for at least 1 week before the barbotage. Thus, this case is significant in providing support for ultrasound-guided barbotage for patients who do not respond to conservative medical management. Patients of any age who endorse severe pain at rest or ambulation would benefit from this therapy.

Furthermore, it is important to utilize a systematic approach to the clinical presentation due to high misdiagnosis rates for infective or inflammatory pathophysiology, arthropathies, or neoplasia [20–22]. Diagnosis, including gout, pseudogout, or infectious etiologies such as osteomyelitis, must be considered in acute ankle pain presentations. Laboratory tests such as C-reactive protein, complete blood count, erythrocyte sedimentation rate, and diagnostic imaging such as MRI or US should be considered [23]. Although our case lacked laboratory testing due to an emergent presentation, it is imperative to consider all differentials.

## Conclusion

While it is a relatively new discovery, the Gruberi bursa does contain inflammatory components that predispose it to calcification, as presented in this case. Further research is needed to determine the pathophysiology of calcification in the talonavicular region. Additionally, studies should be performed to identify inclusion criteria and compare the efficacy of ultrasound-guided barbotage to other treatments of calcific Gruberi bursitis. This report demonstrates the value of enhanced MRI and significant improvement using ultrasound-guided barbotage after failed conservative medical management.

## Data Availability

The data and materials supporting the findings of this study are available upon reasonable request from the corresponding author.

## Abbreviations

EDL: Extensor digitorum longus
AP: Anteroposterior
MRI: Magnetic resonance imaging
US: Ultrasound
